# Resection and survival data from a clinical trial of glioblastoma multiforme‐specific IRDye800‐BBN fluorescence‐guided surgery

**DOI:** 10.1002/btm2.10182

**Published:** 2020-08-31

**Authors:** Kunshan He, Chongwei Chi, Deling Li, Jingjing Zhang, Gang Niu, Fangqiao Lv, Junmei Wang, Wenqiang Che, Liwei Zhang, Nan Ji, Zhaohui Zhu, Jie Tian, Xiaoyuan Chen

**Affiliations:** ^1^ Beijing Advanced Innovation Center for Big Data‐Based Precision Medicine Beihang University Beijing China; ^2^ CAS Key Laboratory of Molecular Imaging, Institute of Automation Chinese Academy of Sciences Beijing China; ^3^ Department of Neurosurgery, Beijing Tiantan Hospital Capital Medical University Beijing China; ^4^ China National Clinical Research Center for Neurological Diseases (NCRC‐ND) Beijing China; ^5^ Department of Nuclear Medicine, Peking Union Medical College Hospital Chinese Academy of Medical Sciences and Peking Union Medical College Beijing China; ^6^ Laboratory of Molecular Imaging and Nanomedicine (LOMIN) National Institute of Biomedical Imaging and Bioengineering (NIBIB), National Institutes of Health (NIH) Bethesda Maryland USA; ^7^ Department of Cell Biology, School of Basic Medical Sciences Capital Medical University Beijing China; ^8^ Department of Neuropathology, Beijing Neurosurgical Institute Capital Medical University Beijing China

**Keywords:** fluorescent IRDye800‐BBN, glioma, intraoperative, neurosurgery, prognosis

## Abstract

Supra‐maximum surgical tumor resection without neurological damage is highly valuable for treatment and prognosis of patients with glioblastoma multiforme (GBM). We developed a GBM‐specific fluorescence probe using IRDye800CW (peak absorption/emission, 778/795 nm) and bombesin (BBN), which (IRDye800‐BBN) targets the gastrin‐releasing peptide receptor, and evaluated the image‐guided resection efficiency, sensitivity, specificity, and survivability. Twenty‐nine patients with newly diagnosed GBM were enrolled. Sixteen hours preoperatively, IRDye800‐BBN (1 mg in 20 ml sterile water) was intravenously administered. A customized fluorescence surgical navigation system was used intraoperatively. Postoperatively, enhanced magnetic resonance images were used to assess the residual tumor volume, calculate the resection extent, and confirm whether complete resection was achieved. Tumor tissues and nonfluorescent brain tissue in adjacent noneloquent boundary areas were harvested and assessed for diagnostic accuracy. Complete resection was achieved in 82.76% of patients. The median extent of resection was 100% (range, 90.6–100%). Eighty‐nine samples were harvested, including 70 fluorescence‐positive and 19 fluorescence‐negative samples. The sensitivity and specificity of IRDye800‐BBN were 94.44% (95% CI, 85.65–98.21%) and 88.24% (95% CI, 62.25–97.94%), respectively. Twenty‐five patients were followed up (median, 13.5 [3.1–36.0] months), and 14 had died. The mean preoperative and immediate and 6‐month postoperative Karnofsky performance scores were 77.9 ± 11.8, 71.3 ± 19.2, and 82.6 ± 14.7, respectively. The median overall and progression‐free survival were 23.1 and 14.1 months, respectively. In conclusion, GBM‐specific fluorescent IRDye800‐BBN can help neurosurgeons identify the tumor boundary with sensitivity and specificity, and may improve survival outcomes.

## 1 INTRODUCTION

Glioblastoma multiforme (GBM) is considered a grade IV malignant glioma according to the World Health Organization (WHO) and is among the malignancies with the worst prognoses. No cure is presently available, and affected patients have a median overall survival (OS) of 14.6 months after treatment with a standard combination of surgery, radiation therapy, and chemotherapy.^1^, ^2^, ^3^

A maximum safe tumor resection is the current goal in the treatment of GBM.^4^, ^5^, ^6^ However, this goal is extremely difficult to achieve because characteristically, GBM is diffusely infiltrating and highly proliferative and generally does not display a location preference.^7^ Additionally, more than 50% of GBM tumors are located near or within the eloquent areas of the brain.^8^ Damage to a functionally eloquent area can cause inevitable postoperative neurological deficits, including motor weakness, sensory deficits, language difficulties, and visual deficits.^9^ During the past two decades, advanced neurosurgical imaging technologies, such as neuronavigation,^10^, ^11^ intraoperative ultrasonography (iUS),^12^, ^13^ and intraoperative magnetic resonance imaging (iMRI),^14^, ^15^ have been developed to achieve the maximum degree of tumor resection without incurring neurological deficits. Although these technologies have improved the potential to achieve a complete resection of GBM, all are associated with limitations and technical issues.^16^ For example, iMRI may cause image distortion and inaccurate target registration,^17^ and imaging artifacts that arise during iUS guidance could reduce the tumor detection sensitivity and specificity.^18^ Moreover, a residual disease measuring <1 cm in diameter might be missed by iUS.^19^

Optical fluorescence imaging is a cost‐effective and time‐efficient alternative technique that can be used during GBM resection. 5‐Aminolevulinic acid (5‐ALA), a natural biochemical precursor of hemoglobin, can elicit the synthesis and accumulation of fluorescent porphyrins in malignant glioma tissue. A Phase III trial verified that 5‐ALA enables a more complete resection of tumors and has led to improvements in 6‐month progression‐free survival (PFS) outcomes.^20^ Fluorescein sodium (FS) is a fluorophore that, when intravenously injected, accumulates selectively in high‐grade glioma (HGG) cells via an altered blood–brain barrier (BBB). A Phase II study reported that FS injection is both safe and feasible and enables a high rate of complete resection.^21^ However, both 5‐ALA and FS lack active targeting capabilities and are characterized by limited penetration depths, strong background fluorescence, and autofluorescence. Near‐infrared fluorescent (NIRF) dyes, which have excitation and emission wavelengths between 700 and 900 nm, could be used to overcome these negative effects.^22^, ^23^

Gastrin‐releasing peptide receptor (GRPR), also known as bombesin (BBN) receptor subtype II, is overexpressed in multiple tumor types, including glioma.^24^ We previously verified the feasibility and safety of BBN conjugated to the NIRF dye IRDye800CW (IRDye800‐BBN) during GBM surgery.^25^ In this study, we aimed to determine the resection efficiency, sensitivity, specificity, and survivability of IRDye800‐BBN‐assisted neurosurgery for patients with GBM.

## 2 MATERIALS AND METHODS

### 2.1 Patients

Patients with preoperative enhanced MRI imaging and/or pathological evidence of a newly diagnosed GBM considered suitable for surgical removal were recruited at Peking Union Medical College Hospital and Beijing Tiantan Hospital. The exclusion criteria were as follows: preoperative Karnofsky Performance Status (KPS) score < 70; mental disease; severe liver or kidney illness with a serum creatinine concentration >3.0 mg/dl; any liver enzyme level ≥5× above the normal upper limit; a severe allergy to intravenous radiographic contrast; claustrophobia or an inability to accept positron emission tomography (PET)/computed tomography (CT) or PET/MRI scanning; pregnancy or breast feeding; and an inability to voluntarily provide informed consent.

The tumor locations relevant to the eloquent brain areas were categorized as Grades I, II, and III, indicating noneloquent, near‐eloquent areas, and eloquent areas, respectively. The ethics committee of the Peking Union Medical College Hospital and Beijing Tiantan Hospital approved this study. All included patients provided signed informed consent preoperatively and underwent surgery at Beijing Tiantan Hospital. The clinical trial number of the study is NCT 02910804.

### 2.2 IRDye800‐BBN preparation

**FIGURE 1:**
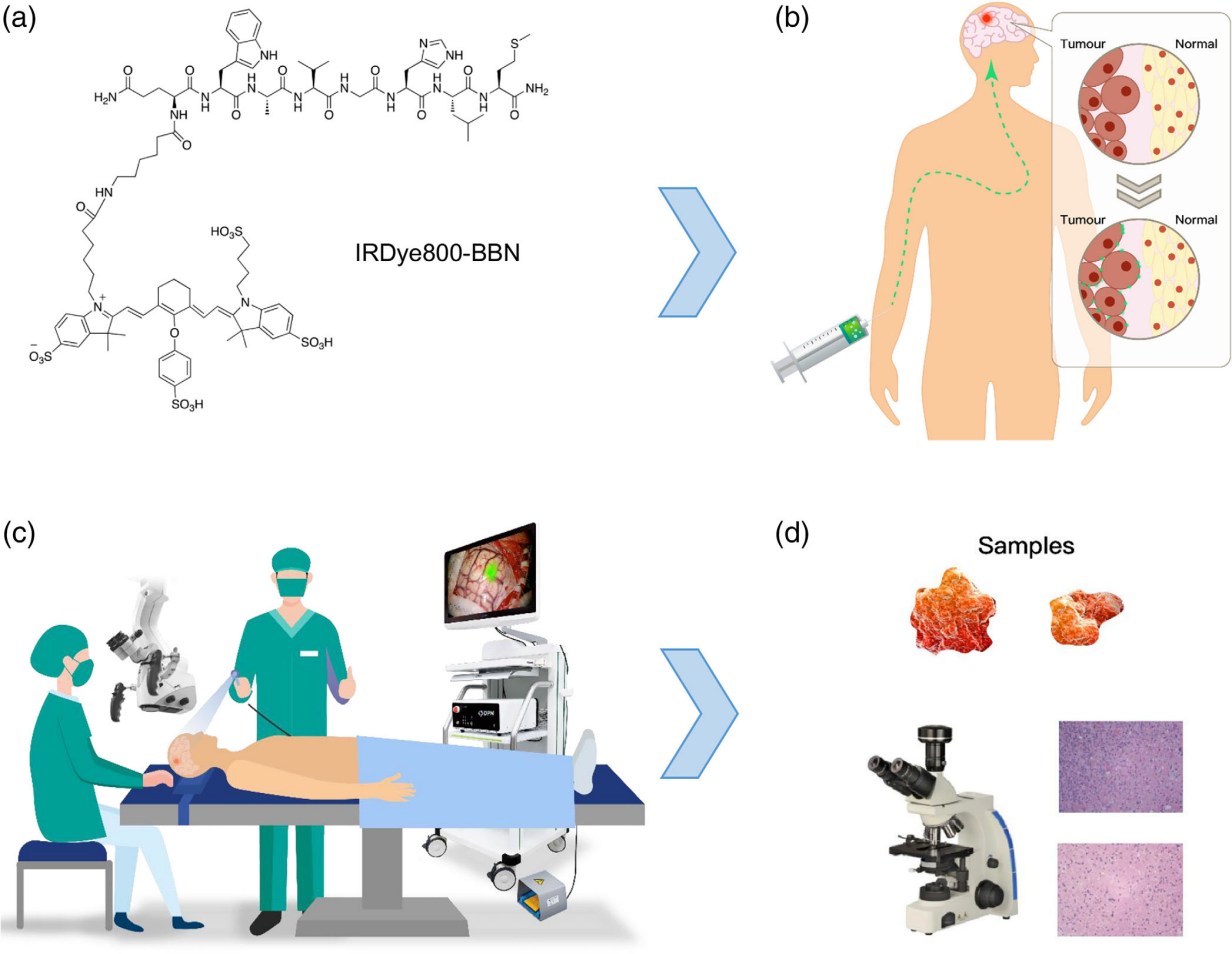
Description of glioblastoma multiforme‐specific fluorescence‐guided surgery using IRDye800‐BBN. (a) IRDye800‐BBN formula. (b) IRDye800‐BBN was intravenously infused 16 hr before induction of anesthesia. (c) After opening the dura, DPM‐III‐01 was used to determine the area and boundary of the tumor. (d) After the operation, pathological examination of the samples was conducted

Clinical‐grade IRDye800‐BBN (Figure 1a) was produced using current good manufacturing practices (cGMP). Briefly, 10.5 mg of BBN and 8.0 mg of IRDye800CW (LI‐COR Biosciences Inc.) were dissolved in 4 ml of dimethyl formamide (DMF) in a 20 ml glass vial, to which 0.05 ml of *N*,*N*‐diisopropylethylamine was added. The mixture was stirred at room temperature for 2 hr and monitored via analytical high‐performance liquid chromatography (HPLC). Once IRDye800CW was completely consumed, the mixture was diluted with 4 ml of water purified on a C‐18 prep‐HPLC in two separate injections with a linear gradient starting from 6% A (0.1% TFA in acetonitrile) and 94% B (0.1% TFA in water) for 5 min; this was increased to 65% A in 35 min at a flow rate of 12 ml/min. The fractions containing the desired product were collected, combined, and lyophilized to give 11.4 mg of final product with an 81.4% yield. The purity of the product was >97% by analytical HPLC with a linear gradient starting from 5% A and 95% B for 5 min; this was increased to 65% A by 35 min at a flow rate of 1 ml/min. The identity of the product was verified by LC–MS: [(MHH)/2]^++^ = 1,017.8367 (m/z), calc: 2036.7891 (C_95_H_128_N_16_O_24_S_5_).

### 2.3 Fluorescence imaging system

We developed a customized imaging system (DPM‐III‐01, Zhuhai Dipu Medical Technology Co., Ltd.) based on the fluorescence properties of IRDye800‐BBN.^26^, ^27^, ^28^ We designed a new 778 nm laser and re‐optimized the optical path and optical elements to capture the emission fluorescence signal at 795 nm with maximum efficiency. The DPM‐III‐01 system could simultaneously acquire white light images while performing NIR imaging with an overlaid imaging capability. In addition, it can convert NIR images into heat maps.

### 2.4 Surgical protocol

IRDye800‐BBN was infused intravenously at a dose of 1 mg in 20 ml of sterile water and was administered 16 hr before the induction of anesthesia (Figure 1b).^25^ Neuronavigation was allowed only for the incision, bone flap, and cortex incision region, but not while resecting the tumor or dissecting the residual tumor around the tumor cavity. During GBM resection, the DPM‐III‐01 system was used to determine the extent of resection. Other techniques, such as iUS and iMRI, were not utilized.

After opening the dura, the DPM‐III‐01 was used to determine the area and boundary of the tumor (Figure 1c). If IRDye800‐BBN fluorescence was present in a safe area, the tumor was resected using a white‐light microscope (M205FA, Leica, Germany). The two processes were alternated until wound bed was reached. Finally the tumor cavity was re‐examined using the DPM‐III‐01. If additional fluorescence was detected in a safe area, the tissue was further resected and the specimens from different locations were submitted for neuropathological analysis. When safe and feasible, some biopsies were randomly harvested from nonfluorescent brain tissue in noneloquent areas around the tumor cavity to assess the diagnostic accuracy. To avoid inter‐surgeon variability, all the operations were performed by the same neurosurgeon, who had 25 years of experience in brain tumor practice. All biopsies were analyzed according to standard pathological procedures (Figure 1d).

### 2.5 MRI analysis

All the patients were intravenously injected with 0.1 mmol/kg body weight of Magnevist (Omniscan, GE healthcare) and underwent MRI scans on a 3.0T scanner. The slice thickness was set to 5 mm. Preoperative enhanced MRI imaging was performed within 1 week before surgery. Early postoperative MRI scans were performed within 72 hr postoperatively. All MRI data were analyzed in the Department of Neuroradiology at Beijing Tiantan Hospital.

The tumor volume was defined as a high‐signal area after T1‐weighted enhancement and was calculated as follows. The tumor contour of each slice was segmented by two experienced neuropathologists using Picture Archiving and Communication Systems (PACS) and was then cross‐verified. If discrepancies arose, a consensus opinion was obtained from another higher‐level pathologist. The areas on each slice were added, and this sum was multiplied by the thickness.

The extent of resection was calculated as: (preoperative tumor volume—postoperative tumor volume)/preoperative tumor volume × 100%. The completeness of tumor resection was defined as a residual tumor volume of <0.175 cm^3^, in accordance with previous studies.^29^, ^30^ Neuropathologists were blinded to the IRDye800‐BBN fluorescence‐guided surgery when calculating the completeness of tumor resection.

### 2.6 Follow‐up

The patients' living conditions were assessed using the KPS. PFS was defined as the time interval between surgical treatment and the first appearance of disease progression. Disease progression was defined as evidence of residual tumor volume growth in a patient with an incomplete resection or the presence of new lesions in a patient with complete resection, according to the Response Assessment in Neuro‐Oncology (RANO) criteria. The endpoint was death from any cause. OS was defined as the time interval between surgical treatment and death from any cause. The adverse events experienced by patients were documented according to the Common Terminology Criteria for Adverse Event (CTCAE) guidelines, and the relationship between these events and the use of IRDye800‐BBN was analyzed.

### 2.7 Statistical analysis

The signal‐to‐background ratio (SBR) of the IRDye800‐BBN fluorescence region to the peripheral brain parenchyma (PBP) was calculated as follows. ImageJ software (Version: 1.52v, National Institutes of Health, Bethesda, MD) was first used to plot a region of interest (ROI) in the fluorescent region, and the mean gray value of the ROI (V_Signal_) was calculated. Subsequently, a same‐sized ROI was placed in the PBP, and the mean gray value (V_Background_) was calculated. The SBR values of each participant in the study were measured five times by dividing the V_Signal_ by the V_Background_.

Perioperative data are represented using the usual descriptive statistical methods: mean, median, and standard deviation (SD) for continuous variables and whole numbers and percentages for categorical variables. Tukey's multiple comparison test was applied to compare the KPS values over time (preoperative, immediate and 6‐month postoperative). A repeated measures analysis of variance and a posthoc test based on the tumor eloquence groupings were performed to determine whether the KPS scores differed across the groups. The log‐rank test was used to determine the relationship between OS, methylguanine‐DNA methyltransferase (*MGMT*) promoter methylation, and the isocitrate dehydrogenase (*IDH*) gene status. A *p*‐value <.05 was considered to indicate statistical significance. The sensitivity and specificity were calculated using the 2015 Standards for Reporting Diagnostic Accuracy (STARD).^31^ Both the neuroradiologists and neuropathologists were blinded to the survival data and IRDye800‐BBN fluorescence data.

## 3 RESULTS

### 3.1 Patient characteristics

From April 2016 to August 29, 2018 patients who met the inclusion criteria participated in this study. Their median age was 54 (range, 17–70) years, and their median preoperative KPS score was 80 (range, 70–100). Regarding tumor locations, 8, 9, and 12 tumors were classified as Grade I, II, and Grade III, respectively. The patients' characteristics are described in Table 1.

**TABLE 1:** Patient characteristics

Variables	Values
No. of enrolled patients	29
Age (years)	
Median	54
Range	17–70
Preoperative KPS score	
Median	80
Range	70–100
Preoperative contrast‐enhanced tumor volume (cm^3^)	
Mean	34.10
Median	29.51
Range	2.75–87.76
Eloquence	
Grade I (noneloquent)	8
Grade II (near‐eloquent)	9
Grade III (eloquent)	12
Extent of resection	
Median	100%
Range	90.6–100%
OS (months)	
Median	23.1
PFS (months)	
Median	14.1
IDH	
Wild‐type	15
Mutant	10
MGMT	
Methylated	16
Unmethylated	9

Abbreviations: IDH, isocitrate dehydrogenase; KPS, Karnofsky performance status; MGMT, methylguanine‐DNA methyltransferase; OS, overall survival; PFS, progression‐free survival.

### 3.2 Fluorescence characteristics

**FIGURE 2:**
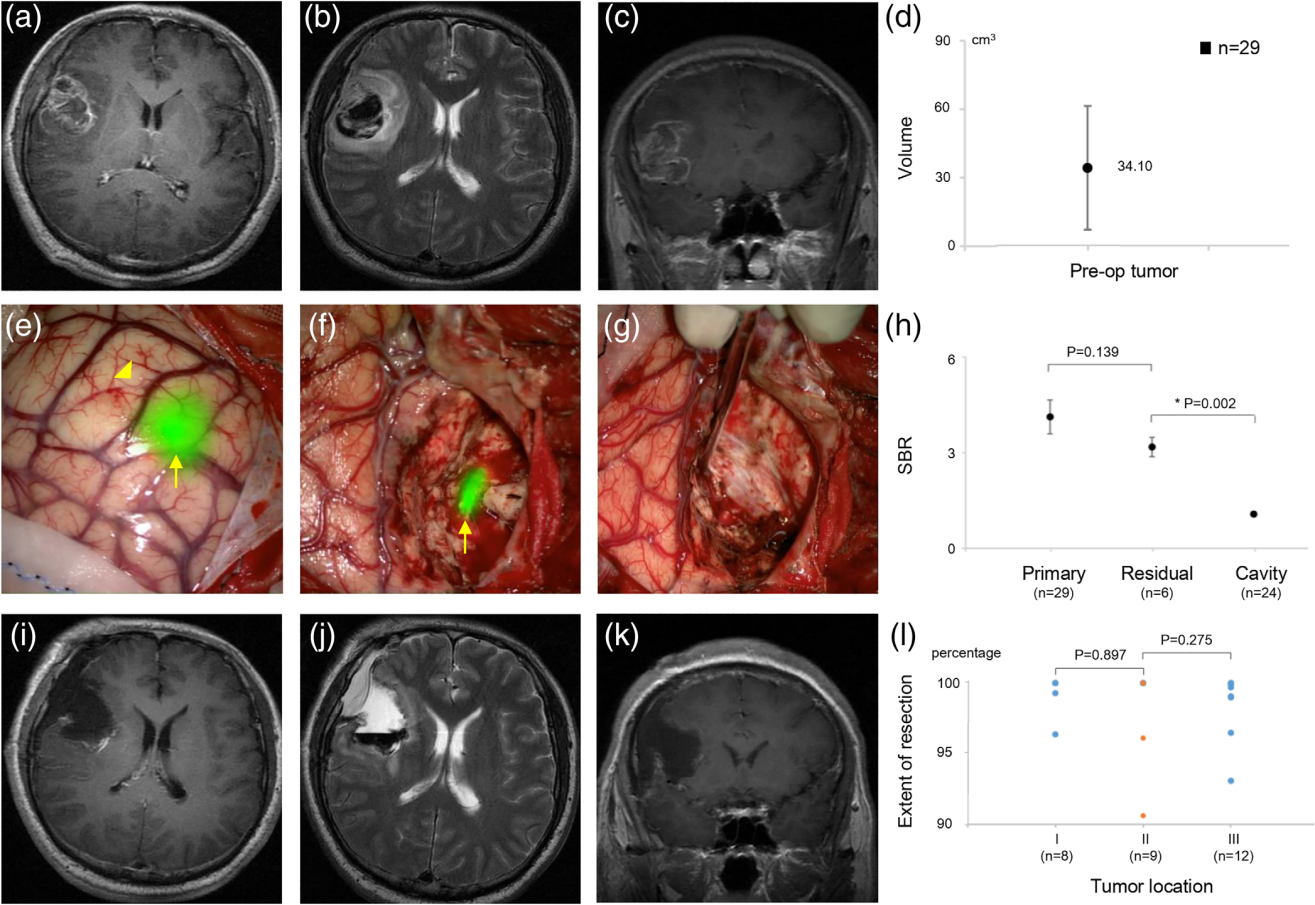
Preoperative, intraoperative, and postoperative imaging data of fluorescence‐guided surgical resection of a glioblastoma multiforme (GBM) with IRDye800‐BBN. (a–c) Reprehensively preoperative enhancing brain magnetic resonance imaging (MRI) of a right frontal GBM. (d) Preoperative contrast‐enhanced tumor volume. (e) Immediately after opening the dura, the tumor (arrow) showed an obvious fluorescence; the normal cortical area and superficial vein (arrowhead) showed no fluorescence. (f) The residual tumor (arrow) showed an obvious fluorescence. (g) The tumor cavity did not exhibit any obvious fluorescence, indicating a complete resection. (h) The signal‐to‐background ratio (SBR) values of the primary tumor, residual tumor, and tumor cavity. (i–k) Postoperative MRI confirmed a lack of residual tumor. (l) Extent of resection in different tumor locations

Fluorescence‐guided surgery (FGS) with IRDye800‐BBN was successfully performed on all enrolled patients. The recruited subjects were treated with FGS according to the preoperative enhanced brain MRI diagnoses (Figure 2a–c). The mean preoperative contrast‐enhanced tumor volume was 34.10 (range, 2.75–87.76) cm^3^ (Figure 2d). Immediately after opening the dura, the superficial tumor exhibited obvious fluorescence (arrow). In contrast, the normal cortical area and superficial vein (arrowhead) showed no fluorescence due to the sufficient washout time (Figure 2e). However, for deep‐seated tumors, fluorescent signals could only be detected following sufficient exposure. The mean SBR value of all fluorescent tumor regions was 4.24 ± 0.53, which allowed neurosurgeons to differentiate tumor tissues from PBP sufficiently during surgery. The strong fluorescence signal lasted long enough to complete the tumor resection. After debulking most of the tumor, any fluorescence remaining in the tumor cavity indicated the residual tumor (arrow), with an SBR value of 3.17 ± 0.30 (Figure 2f). Further resection was terminated when a tumor cavity without any obvious fluorescence, with an SBR value of 1.18 ± 0.08 (Figure 2g). The relationship between the above three SBR values is shown in Figure 2h.

Postoperative MRI demonstrated that a total tumor resection was achieved in the representative case (Figure 2i–k). Complete resection was achieved in 24 of 29 included patients (82.76%) (Figure 2l); of the remaining 5 patients, 2 had Grade II tumors near eloquent areas such as the insular lobe, and 2 had Grade III tumors located in eloquent areas such as the precentral gyrus and pontine areas. Intraoperative electrophysiological monitoring showed that further resection of these lesions would damage specific functions, and the operations were stopped despite the remaining fluorescence‐emitting tissues in the cavities. Notably, one patient had residual tumors that did not fluoresce intraoperatively, leading to incomplete tumor resection. Because GBM tumors are highly infiltrative, the further away from the tumor core or bulk, the more infiltrative cells are present. Thus, there may still be tumor cells present without fluorescence in the glial hyperplasia area, which is intraoperatively regarded as the tumor boundary.

### 3.3 Sensitivity and specificity

**FIGURE 3:**
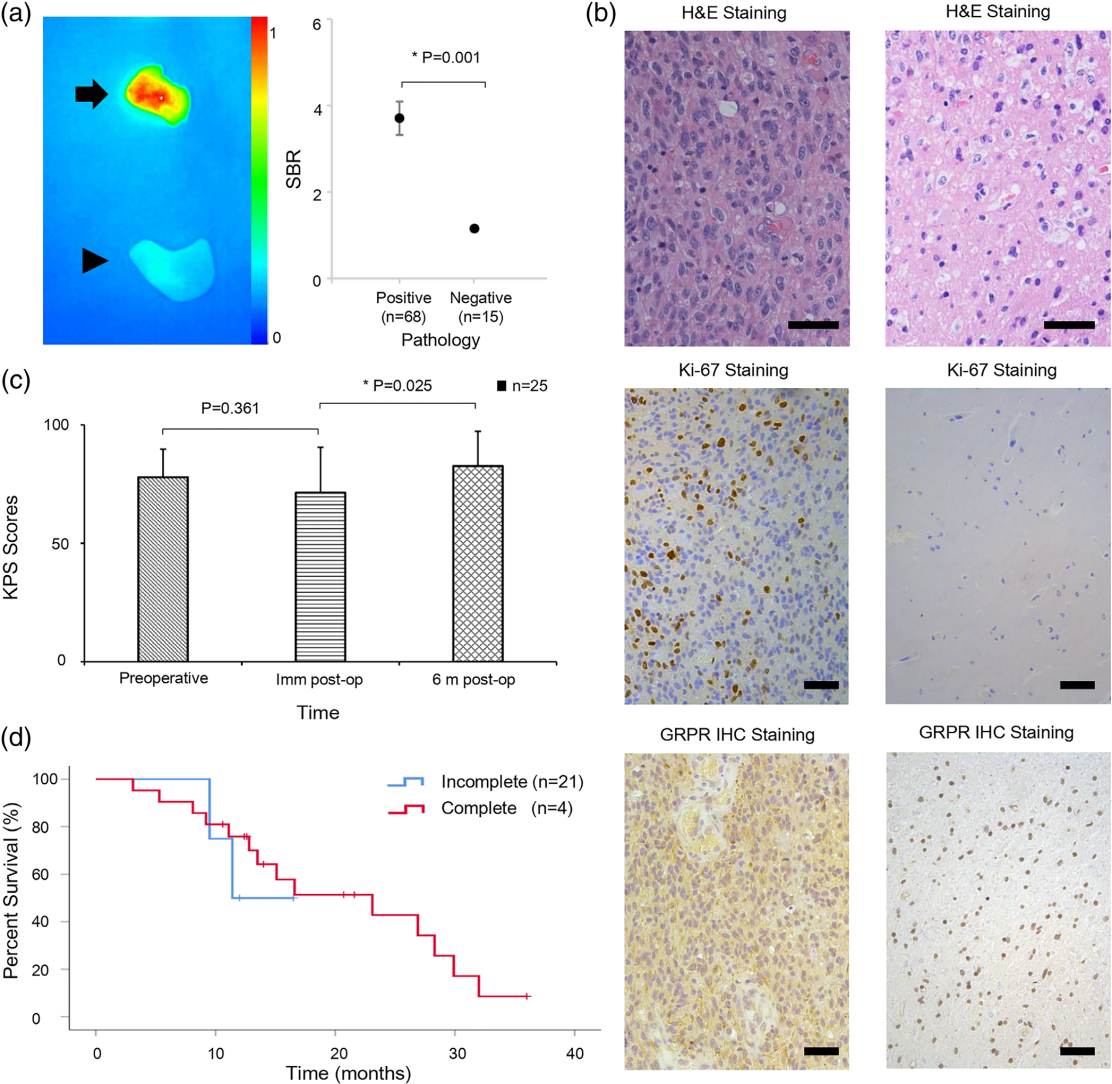
Pathology results and patients' Karnofsky performance status (KPS) scores and survival curve. (a) In the left subgraph, fluoresce profile of a representative true positive specimen (arrow) and true negative specimen (arrowhead); in the right subgraph, SBR values of true positive specimens and true negative specimens of all patients. (b) H&E, Ki‐67 and GRPR IHC staining results of specimens in panel A. Scale bar, 50 μm. The positive specimen was confirmed by H&E and Ki‐67 staining and had a large number of GRPR positive cells (left column), while the negative specimen only had a few (right column). (c) Preoperative, immediate postoperative, and 6‐month postoperative KPS scores. (d) Kaplan–Meier plots of overall times stratified by extent of tumor resection

Eighty‐nine samples were harvested at the tumor margin, including 70 fluorescence‐positive and 19 fluorescence‐negative samples (Table 2). Of the 70 biopsies harvested from IRDye800‐BBN fluorescent regions, 68 showed evidence of a GBM tumor. Of the 19 biopsies in the nonfluorescent regions from 19 patients, 15 showed no confirmed GBM tumor. The SBR value of true positive specimens was 3.71 ± 0.39, while that of true negative specimens was 1.15 ± 0.06 (Figure 3a). The resulting sensitivity and specificity of IRDye800‐BBN for identifying GBM tumors were 94.44% (95% confidence interval [CI] 85.65–98.21%) and 88.24% (95% CI 62.25–97.94%), respectively. Consequently, the median gross total resection (GTR) of all GBM patients was 100% (range, 90.6–100%). Postoperative pathological results showed that the positive specimens had a large number of GRPR positive cells while the negative specimens only had a few (Figure 3b).

**TABLE 2:** Pathologically confirmed glioblastoma multiforme (GBM) and non‐GBM biopsies according to intraoperative fluorescence

	Pathology‐positive	Pathology‐negative	Total
Fluorescence‐positive	68	2	70
Fluorescence‐negative	4	15	19
Total	72	17	89

### 3.4 Progression‐free survival and OS

No serious adverse reactions associated with IRDye800‐BBN use were reported during the follow‐up. Four of 29 patients, including one patient without complete resection, refused to participate in the follow‐up study because of personal or family reasons. Of the 25 patients who were followed up, 16 exhibited *MGMT* promoter methylation and 15 harbored an *IDH1* mutation. The mean (±SD) preoperative, immediate postoperative, and 6‐month postoperative KPS scores of these patients were 77.9 ± 11.8, 71.3 ± 19.2, and 82.6 ± 14.7, respectively (Figure 3c). The KPS scores did not differ across the tumor eloquence groups (*p* = .311), indicating that patients in Group III had similarly good outcomes as those in Group I. In other words, our method appeared to preserve the functional brain tissue. The median OS was 23.1 months, and the median PFS was 14.1 months. The longest OS exceeded 36 months (Figure 3d), and the longest PFS was 23.8 months. In contrast, the four patients who did not achieve a complete resection had a median OS of only 11.4 months. During follow‐up, 76% (19/25) of patients completed the standard postoperative chemotherapy and radiotherapy.

## 4 DISCUSSION

Our prospective clinical trial demonstrated that FGS of a GBM with IRDye800‐BBN was feasible and effective and could yield a prolonged PFS and OS. During surgery, the specific fluorescence signals emitted by tumor tissues were strong enough for neurosurgeons to discriminate these areas from PBP, lasted long enough to complete the tumor resection, and did not interfere with routine white‐light operative microscopy.

Regarding GBM surgery, increasing evidence indicates that maximal resection could improve the life expectancy of the patient. However, it is crucial to minimize the risk of perioperative morbidity, especially neurological damage.^16^ To circumvent the brain shift and provide the neurosurgeon with surgical information updated real‐time, various advanced intraoperative imaging methods have been developed, including FGS with 5‐ALA or FS. However, these fluorescent agents are neither NIRF dyes nor are actively tumor specific. Compared with visible light, NIR light has a higher SBR value because of the low level of autofluorescence and a greater penetration depth because of the low levels of tissue absorption and scattering.^32^ Therefore, we choose IRDye800CW,^22^ a widely used NIRF dye, to be coupled with BBN for developing a new targeted contrast agent. BBN could bind specifically to GRPR, which is overexpressed in a variety of tumor tissues and is associated with tumor infiltration and growth.^33^ Flores et al found that GRPR was detected in 100% of the glioma samples analyzed, especially in the 24 cases of GBM. Highly expressed GRPR was also observed in tumor endothelial cells. Although GRPR was detected in 10–50% of neuronal cells, it was not found in glial cells from normal brain tissue samples.^34^ Another study also showed that GRPR was not expressed in astrocytes and microglial cells. It was important to intraoperatively differentiate the tumor with the white matter, where only astrocytes and microglial cells existed. Therefore, GRPR was a good and specific target candidate for GBM imaging. In addition, because it is highly specifically targeted to the tumor and uses an intravenous route, the IRDye800‐BBN dose was much lower than the required doses of 5‐ALA and FS for FGS (1 mg per patient vs. 20 mg/kg for 5‐ALA and 5–10 mg/kg for FS).

Although there was no control group, the OS and PFS durations found in this study were significantly higher than those found in other studies that used traditional visible light, 5‐ALA, and FS.^3^ For instance, Stummer et al enrolled 322 patients and randomly assigned them to the visible white or 5‐ALA groups. After a median follow‐up of 35.4 months, the respective median PFS and OS durations were 3.6 and 13.5 months in 131 patients for visible white, and 5.1 and 15.2 months in 139 patients for 5‐ALA, respectively.^20^ Remarkably, our study included 9 patients with Grade II tumors and 12 patients with Grade III tumors, which accounted for 72% of the total study population. The mean preoperative contrast‐enhanced tumor volume was 34.10 cm^3^. In comparison, the corresponding values were 65% and 22.4 cm^3^, respectively, in a study where Acerbi and colleagues used FS, in which the respective median PFS and OS durations were 7 months and 12 months, respectively.^21^ Thus, the possibility that the tumor location and size might yield better results has likely been ruled out, further validating out results. However, other hidden variables may have complicated a direct comparison between the studies, including the surgeon's level of skill and the postoperative care provided. Therefore, these conclusions should be interpreted with scientific accuracy and caution.

We acknowledge that the complete resection of a GBM tumor is not always feasible, even when using targeted IRDye800‐BBN. Excision should be avoided when a tumor has invaded the functional areas of the brain. However, the FGS method, including 5‐ALA and FS, is not sensitive for low‐grade gliomas because the BBB remains intact, and it is therefore difficult for contrast agents to bind to tumor tissues.^35^ In our present study, all four false negative specimens were pathologically confirmed to be low‐grade components of a secondary GBM pathological analysis confirmed that the two false positive specimens were from the reactive gliosis zone where the density of cancer cells was very low. However, the BBB might have been partially damaged. Therefore, IRDye800‐BBN was found in tissues where other fluorophores, such as 5‐ALA, were also found. Moreover, we found that patients in the *IDH1* mutation subgroup had a median OS of 26.9 months, whereas those in the *IDH1* wild‐type subgroup had a median OS of 13.5 months. Patients in the methylated *MGMT* subgroup had a median OS of 26.9 months, compared to 12.8 months in the unmethylated MGMT subgroup. However, our analysis revealed no statistical effect of the *MGMT* promoter (*p* = .077) or *IDH1* gene status (*p* = .289) on OS, although this may be due to our small sample size. Therefore, the main limitations of this study were the small sample size and lack of a control group with randomization. Given our current research progress, we will perform a randomized controlled trial of this method versus routine clinical operations using white‐light microscopy or other fluorescent dyes (e.g., 5‐ALA).

## 5 CONCLUSIONS

This study indicated that GBM‐specific NIRF IRDye800‐bombesin can help neurosurgeons sensitively and specifically identify the tumor boundary for complete resection, which may improve survival outcomes. FGS with targeted contrast agents can also be applied in different diseases to benefit patients.

## 6 CONFLICT OF INTEREST

The authors have no conflict of interest to declare.
